# POWIFF- Prospective study of wrist internal fixation of fracture: A protocol for a single centre, superiority, randomised controlled trial to study the efficacy of the VRP (2.0) distal radius plate (Austofix) versus the VA-LCP (Depuy-Synthes) for distal radius fractures

**DOI:** 10.1186/s12891-018-2052-4

**Published:** 2018-04-30

**Authors:** V. D. Varghese, Peter Smitham, Stuart Howell, Suzanne Edwards, Mark Rickman

**Affiliations:** 1 Discipline of Orthopaedics and Trauma, Royal Adelaide Hospital, University of Adelaide, Adelaide, Australia; 2 Adelaide Health Technology Assessment (AHTA), School of Public Health, The University of Adelaide, Adelaide, Australia

**Keywords:** Distal radius fracture, Volar plating, Functional outcome

## Abstract

**Background:**

Distal radial fractures are one of the most common orthopaedic cases that present to the A&E department. Surgical intervention is warranted in displaced intraarticular fractures and fractures with more than the recommended angulation or shortening, and is most commonly treated with volarly placed fixed angle locking plates. The aim of this study is to determine and compare the efficacy of two different plates for surgical treatment of distal radius fractures. The VRP 2.0 is a new plate produced by the Austofix company and this system will be compared against the VA-LP (Variable angle-locking plate) produced by Depuy-Synthes which has been used as the standard treatment device.

**Methods and Design:**

Patients between the ages of 18 and 80 presenting to the Royal Adelaide Hospital with isolated closed distal radial fractures will be invited to participate in this study. A total of 200 patients are required to provide 90% statistical power at a 5% alpha level to detect a difference of 11.5 points on the PRWE (Patient rated Wrist evaluation) score. The primary outcome measure will be the PRWE score while the secondary outcome measures will include the DASH score, EQ5D score, clinical range of movements, grip strength as well as patient perceived return of function at the wrist and time to resumption to work. These will be measured at 6 weeks, 3 months and 12 months. Radiographic indices including the radial tilt, length, volar inclination and plate prominence will also be measured. Complications will be recorded up to 12 months. Post hoc comparisons will be done using paired t tests. An intention to treat and a per protocol analysis will be done to compare the 2 groups.

**Discussion:**

Distal radial fractures are increasingly being treated by internal fixation using volar locking plates. However, there is no prospective study to date comparing one plate against another in terms of outcome and complications. This study could provide more information about the best way to treat these injuries surgically.

**Trial registration:**

The trial is registered with the Australia New Zealand Clinical Trials Registry (ANZCTR). Trial registration date-17/11/2016. Trial registration number-ACTRN12616001590459.

**Electronic supplementary material:**

The online version of this article (10.1186/s12891-018-2052-4) contains supplementary material, which is available to authorized users.

## Background

Distal radial fractures account for 18% of the cases that present to A&E and are more common in women above the age of 60 [1]. However, there is no consensus within the literature on their management [2]. This is at least in part due to the number of factors which have to be considered when managing these injuries, including age of the patient [3], functional requirements and velocity of the injury. Surgeon [4] and fracture configuration related factors [5] further complicate the treatment algorithm. Operative and nonoperative methods of treatment have been shown to have good results [6].

Surgical management is usually opted for if there is shortening of the radius after closed reduction and splinting of more than 3 mm, dorsal radial tilt of more than 10 degrees and an intra articular step of more than 2 mm [7]. Surgical modalities include, percutaneous K wire fixation, external fixation and, open reduction and internal fixation(ORIF) with an increasing trend recently for surgical management by ORIF using volar fixed angle plates [8, 9]. The advantages of volar fixed angle plates include anatomic fixation, early mobilisation and improved grip strength. The argument for ORIF in the elderly group is that it helps them to keep their level of independence [10] and in the younger patients it allows earlier return to activities [11, 12]. While there are no clinical trials comparing different volar fixed angle devices, there are several biomechanical studies comparing screws versus pegs in fixed angle locking plates [13, 14] as well as various volar fixed angled plates [15]. There are also retrospective follow up studies suggesting that if plates with a lower profile design are used [16] and placed proximal to the watershed line [17], this may result in fewer complications due to tendon synovitis and rupture. Dorsal tendon rupture, after ORIF has also been recognised to be due to improper surgical technique which can be avoided [18]. However, there are no previous studies or randomised clinical trials comparing one plate against another in terms of outcome and complications.

A variety of outcome measures have been described in previous studies following distal radius fractures and include patient related outcome scores, wrist range of movements, grip strength and radiographic parameters. Using multiple outcome measures gives a composite idea of outcome as recent studies have shown that radiological parameters do not always correlate with functional outcome [19]. The Patient Reported Wrist Evaluation (PRWE) score and Disability Arm Shoulder Hand (DASH) score are commonly used to report outcomes in wrist injuries [19, 20]. However, PRWE score has been recognised as the best score for specifically measuring pain resolution and functional recovery in the injured wrist, whereas the Disability Arm Shoulder Hand (DASH) score measures the total upper limb extremity function [21, 22]. Multiple studies investigating distal radial fractures have used the PRWE as its primary outcome score [23–25]. European Quality of Life 5 Dimension (EQ5D) score is a generic health status measurement that is used as a secondary outcome measure in many orthopaedic conditions [26]. The Austofix Company (Australia) have designed a new plate, the “VRP 2.0”, with the purported advantages of having a lower profile and better fragment specific fixation. It allows a variable fixation angle of 40 degrees (compared to 30 degrees for other systems) which in theory allows more fragment specific fixation, and therefore a more flexible and proximal plate positioning. This in turn may help to reduce soft tissue irritation and possible flexor tendon issues. The Austofix plating system has a universal plate which can be used for both the left and right side which helps to reduce the inventory and it’s also less expensive than the comparative plate. The Synthes (West Chester, Pennsylvania) VA-LCP 2.4 distal radius system has been used extensively for distal radius fractures with good results [27] and has been used at the Royal Adelaide Hospital for the last 5 years. There are no studies comparing these 2 systems prior to this and we plan to compare these plates using the PRWE score as a primary outcome measure and the EQ5D, DASH scores as well as other objective radiological and clinical parameters.

### Aims

This study aims to provide high quality evidence for the safety and effectiveness of surgical treatment of distal radius fractures by comparing 2 different plating systems. The primary outcome measure will be the PRWE (Patient rated Wrist evaluation) score between the two plates with the assumption being that the Austofix plate will have a superior outcome with a minimum difference in the PRWE of 11.5 points. The study will also correlate the final PRWE and DASH scores with each other, document the radiographic scores in terms of restoration of radial tilt, height and inclination, correlate the radiographic and clinical scores and document all complications, both major and minor in both groups.

### Objectives

This study is a two armed randomised controlled trial with a parallel observational study. The objectives of this study are to assess the comparative performance of the two plates in terms of outcome scores specific for the upper limb and general health scores, as well as radiographic indices regarding restoration of normal wrist anatomy, plate prominence as well as complications. Secondary outcome measures include the DASH (Disability of Arm, Shoulder & Hand) score and the EQ5D (EuroQol Five –Dimension) score.

### Null hypothesis

The null hypothesis is that there is no difference in the PRWE scores between the two plates being used for wrist fixation in this trial, at the end of 1 year post fixation.

## Methods

The protocol (V 1.2, dated october16, 2016) was prepared in accordance with the Standard Protocol Items; Recommendations for Interventional Trials (SPIRIT) guidelines. This study is funded by the Department of Orthopaedics & Trauma, Royal Adelaide Hospital. The trial will be conducted in accordance with the regulations of International conference on Harmonisation Good Clinical practice (ICH-GCP); all collaborators will be trained in GCP and the trial will be reported in line with the Consolidated Standards of reporting Trials (CONSORT) statement.

### Setting

The Royal Adelaide Hospital(RAH) is one of two level 1 tertiary centres in South Australia and serves a population of approximately 1.7 million. The orthopaedic department manages approximately 200 cases of distal radius fractures with surgery annually.

### Study design

A single centre two-arm randomised controlled trial with a parallel observational study trial design will be completed at the RAH. The study population will be of adults aged between 18 and 80 presenting to the RAH with a closed distal radius fracture and who are considered for surgical treatment using volar plating. Associated ulna fractures will be noted and managed according to the operating surgeon’s preferences, and similarly the distal radioulnar joint.

### Ethical considerations

Ethical approval has been obtained from the Royal Adelaide Hospital Human Research Ethics Committee, Central Adelaide Health Network (CALHN). Adverse effects if any will be documented and notified to the research ethics committee. All participants with complications will be managed as per in-house management protocols. Participants will be covered by the standard South Australian health indemnity arrangements.

HREC reference number: HREC/16/RAH/373.

CALHN Reference number: R20160909, prior to the commencement of this trial.

### Consent procedures

Recruitment and consenting will be done by a trained research associate who will present the trial and interventions in a consistent and unbiased manner. Eligible patients will be provided with an information sheet and will have time to discuss their concerns prior to enrolment. (Patient Booklet and participation form-Additional file 1).

Inclusion Criterion and exclusion criterion are as shown in Table 1. Patients with isolated injuries in the upper limb were chosen so as to avoid the effect other injuries in the same limb could have and therefore bias the outcome. As most of the questionnaires are self-explanatory and to be filled in by patients themselves, reasonable proficiency in English is a pre-requisite.

**Table 1:** POWIFF trial - Inclusion and exclusion criterion

Inclusion Criterion	Exclusion criterion
1. Age-18-80	1. Patients with concomitant injuries affecting treatment and rehabilitation of the affected arm
2. Pre-operative DASH score of 30 or less	2. Patients with associated neurovascular injuries requiring immediate surgery
3. Isolated closed distal radius fracture with shortening of radius of more than 3 mm, dorsal radial tilt of more than 10 degrees and an intra articular step of more than 2 mm.	3. Patients with associated significant carpal injuries, including scaphoid fractures
	4. Patient unlikely or unhappy to attend for follow up
	5. Patient with limited English proficiency
	6. Patient without cognitive capacity to consent and participate
	7. Patients with impaired upper limb function prior to injury enough to have a DASH score of more than 30 points

### Recruitment

**Fig. 1:**
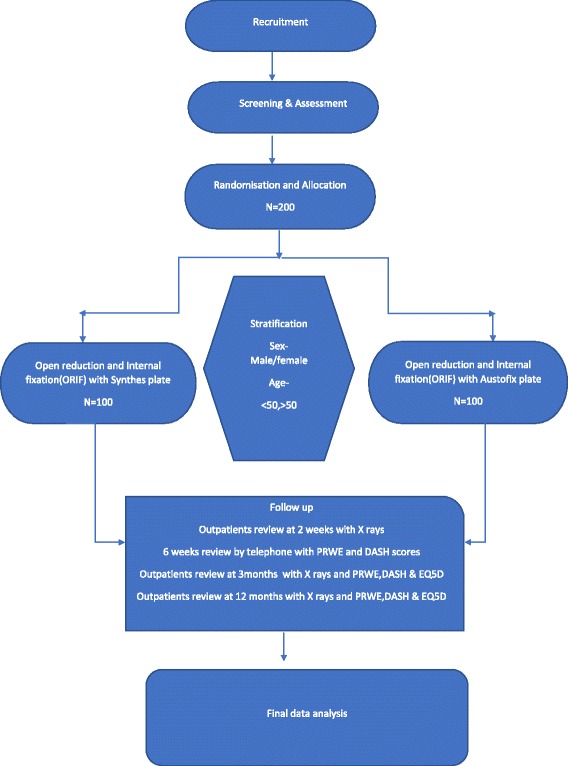
Flow chart showing study protocol, recruitment, randomisation by stratification and final analysis

All patients eligible for the study, and for whom a decision to undergo surgical treatment by volar plating has been made will be invited to participate. Following patient recruitment to the trial, patients will be randomised to receive one or other of the plates. Patients declining randomisation will be managed using the routine plating option, but will still be monitored longitudinally in the standard fashion, forming an observational arm of the study. Inclusion of the patient in the trial will be flagged on their clinical notes by means of a trial sticker. (See Fig. 1). Documentation of complications such as implant failure, revision to another plate system and cross over will be done.

### Randomisation

Allocation of trial treatments will be provided through the local statistics department by a statistician who is not the trial statistician. Randomisation will be a 1:1 allocation using a computer generated randomisation schedule (using Stata Statistical Software: Release 14. College Station, TX: StataCorp LP) stratified by age and sex (four strata) using permuted blocks of size four. A seed was used as the random number generator to specify unique subject identifiers. The patient after being considered eligible for the study and consented by the orthopaedic trainee is stratified according to age and sex. The trainee then informs one of the coinvestigators who obtains the randomisation number from the previously generated schedule. The surgical team will be informed of the plate to be used, once randomised.

### Sample size

Sample size calculations were based on the requirement that effects be assessed at the 5% alpha level with 90% statistical power. It has been shown that the minimum clinically important difference on the PRWE is 11.5 points [28]. Assuming a standard deviation of 20 points, a sample of 65 patients per group would be required; following the application of a variance inflation factor of 1.5 to account for repeated measurements over time and patient attrition, a sample of 98 patients per group would be required.

### Blinding

The patients will be blinded as to which plate they have received until the conclusion of the study. The primary outcome (Patient-rated Wrist Evaluation (PRWE) scores at 12 months) will be collected by blinded researchers, and the statistician will be blinded to the treatment group.

### Interventions

#### Operative management

The specific procedure for each patient managed operatively will be determined by the operating surgeon. Any adverse intra-operative and post-operative event will be recorded. All surgical procedures will be done by one of the senior orthopaedic registrars, trauma fellows or the orthopaedic consultants.

Operative time, plate size, data on the tourniquet time, radiation time and complications if any, and the number of times the metaphyseal screws have been exchanged will be recorded in both groups.

#### Post-operative protocol

Post operatively all patients will be supported in a back slab for 2 weeks, with instructions to commence early and immediate full passive and active ROM exercises of the shoulder, elbow, and hand as allowed by pain. After 2 weeks, the back slab will be removed and full mobilisation allowed, unless injuries to the distal ulna or DRUJ require longer immobilisation. Documentation of associated injuries to the DRUJ (distal radioulnar joint) and treatment will be made. Within this protocol, all patients will follow a standardised shoulder, elbow, and wrist motion and strengthening program outlined in a patient instruction sheet. (See appendix).

The following general reviews will be undertaken:

1. Clinical review at **2 weeks**, with wound check and X-ray
2. Telephone review at 6 weeks with DASH and PRWE scores
3. Clinical review at **3 months** – EQ5D, DASH and PRWE scores
4. Clinical review and Xray at **12 months**. EQ5D, DASH and PRWE scores. Patients will be given follow up appointments at each visit (Fig. 1).

### Outcome measures

#### Primary outcome

The PRWE(Patient rated wrist evaluation score) score will serve as the primary outcome measure, to be taken at 6 weeks, 3 and 12 months post intervention [29]. The PRWE is a well validated, self-report questionnaire used in studies of wrist function / injuries, which gives information on pain and function of the wrist joint [30, 31]. It consists of a 15-item questionnaire designed to measure a patient’s wrist pain and disability. It has two subscales (pain and function) and has a score range from 0 (no disability) to 100 (severe disability).In order to evaluate effects of treatments, a minimum difference of 11.5 points is required to signify clinically significant difference [28]. The final PRWE score at the end of 1 year will be used to assess any difference between the 2 groups.

#### Secondary outcomes

1. The DASH score is to be collected as a secondary outcome, in order to assess function of the whole upper limb, at 6 weeks, 3 months and one year [32]. The DASH score has a range from 0 to 100 with higher scores representing greater disability. Subjects rate task difficulty regardless of hand dominance or affected side, thus reflecting their total upper-extremity disability. Both the DASH and PRWE scores have been shown to be ideal and specific as a research tool for the upper limb [30]. The final DASH score at the end of 1 year will be used for definitive comparison between the 2 groups.
2. The EQ5D (EuroQol) score will also be collected as a secondary outcome measure, at 3 months and one year. This score is a validated measure of health related quality of life, consisting of a 5 health domains as well as a separate VAS (Visual analogue) score. The five domains included in the EQ-5D are: mobility; self-care; usual activities; pain/discomfort and anxiety/depression. The EQ5D is the measure that tends to be most utilised in cost utility analysis [33] . The final EQ5D at the end of 1 year will be used for definitive analysis.
3. Complications (including surgical site infection, unplanned surgery, implant failure, loss of reduction/screw breakout or implant fracture and post-operative nerve palsy up to one year) in both groups will also be documented and compared.
4. Surgical data including operation time and screw numbers
5. Radiographic indices including radial height, tilt and inclination and rates of non-union / delayed union and the Soong score will be analysed and compared in both groups.
6. Soong score will be used to assess prominence and profile of the plate [17].
7. The active range of motion (ROM)at the wrist and grip strength at 3 months and one year will be measured.
8. Time to resumption of work and patient perceived percentage return of global function at 6 weeks, 3 months and 1 year.

### Data management

The case report forms will be designed by the trial coordinator in conjunction with the trial management team. All patient identifiable information will be held in accordance with the GCP (Good clinical practice) guidelines. Participants will be identified by a trial number and a hospital ID number. The deidentified data will be entered in an electronic format by the research assistant in an EXCEL format, who will also coordinate the follow up visits and collection of the patient filled data forms. The data will be kept in a secure electronic form on a password protected hospital computer, and will be destroyed 15 years after the completion of the study.

### Statistical analysis plan

Continuous measures will be summarised as means with standard deviations and medians with inter-quartile range. Categorical measures will be summarised as percentages. Treatment effects will be assessed over time using linear mixed effects models with patient treated as a random factor. A normal distribution with an identity link function will be assumed for continuous measures, while a multinomial distribution and cumulative logit function will be applied to ordinal outcomes. An intention-to treat and a per-protocol analysis will be considered. All tests will be two-tailed and assessed at the 5% alpha level.

### Trial organisation, regulation and oversight

All the cases will be individually reviewed as part of the daily trauma meeting held in the department. All issues pertaining to the management of the trial will be coordinated by the trial management group which will consist of the chief investigator, coinvestigators, trial manger, statistician and the department head. The data management committee will review the trial progress, interim data and safety aspects of the study at 6 months and at completion. The allocated recruitment period for the trial is 24 months. Recruitment will begin in March 2017 and is due to end in March 2019 or until 200 patients are recruited, with a further 1 year period for follow up, data analysis and manuscript submission.

### Dissemination

The results of the study will be submitted for consideration of publication in full in a peer-reviewed journal. Additionally, results will be presented at national and international orthopaedic scientific meetings such as the Australian Orthopaedic Association Annual Scientific Meeting (AOA ASM) and the American Academy of Orthopaedic Surgeons Annual Scientific Meeting. The results will be made available to participants, participating institutions and the media.

### Funding and sponsorship

This study will present independent research funded by the Department of Orthopaedics and Trauma, Royal Adelaide Hospital. This trial is not funded by either of the two companies whose implants are being assessed.

## Discussion

Distal radial fractures are increasingly being treated by internal fixation using volar locking plates. Whilst the benefits of internal fixation are widely accepted for varying groups of patients, there are few prospective studies to date comparing one plate against another in terms of outcome and complications. This study should provide more information about the overall outcomes of this injury by presenting outcome data from large group of surgically managed patients. In addition, new information will be identified through the direct comparison of 2 different fixation devices.

## Additional file

Additional file 1: Patient booklet and consent form with information sheet. (DOCX 49 kb)

## References

[1] Court-Brown CM, Caesar B (2006). Epidemiology of adult fractures: A review. Injury.

[2] Handoll HHG, Huntley JS, Madhok R (2007). External fixation versus conservative treatment for distal radial fractures in adults. Cochrane Database Syst Rev.

[3] Chung KC, Squitieri L, Kim HM (2008). A Comparative Outcomes Study Using the Volar Locking Plating System for Distal Radius Fractures in both Young Adults and Adults Older than 60 Years. The Journal of hand surgery.

[4] Ward CM, Kuhl TL, Adams BD (2011). Early complications of volar plating of distal radius fractures and their relationship to surgeon experience. Hand (N Y).

[5] Thorninger R, Madsen ML, Waever D, Borris LC, Rolfing JHD (2017). Complications of volar locking plating of distal radius fractures in 576 patients with 3.2 years follow-up. Injury.

[6] Kreder HJ, Agel J, McKee MD, Schemitsch EH, Stephen D, Hanel DP (2006). A randomized, controlled trial of distal radius fractures with metaphyseal displacement but without joint incongruity: closed reduction and casting versus closed reduction, spanning external fixation, and optional percutaneous K-wires. J Orthop Trauma.

[7] Lichtman DM, Bindra RR, Boyer MI, Putnam MD, Ring D, Slutsky DJ, Taras JS, Watters WC, Goldberg MJ, Keith M (2011). American Academy of Orthopaedic Surgeons clinical practice guideline on: the treatment of distal radius fractures. J Bone Joint Surg Am.

[8] Gallacher PD, Gilbert R, Memon S, Bhoora IG (2009). Volar plating of distal radius fractures using a locked anatomically contoured plate. Eur J Orthop Surg Traumatol.

[9] Mellstrand-Navarro C, Pettersson HJ, Tornqvist H, Ponzer S (2014). The operative treatment of fractures of the distal radius is increasing: results from a nationwide Swedish study. Bone Joint J.

[10] Orbay JL, Fernandez DL (2004). Volar fixed-angle plate fixation for unstable distal radius fractures in the elderly patient. J Hand Surg Am.

[11] Wilcke MKT, Abbaszadegan H, Adolphson PY (2011). Wrist function recovers more rapidly after volar locked plating than after external fixation but the outcomes are similar after 1 year: A randomized study of 63 patients with a dorsally displaced fracture of the distal radius. Acta Orthop.

[12] Drobetz H, Koval L, Weninger P, Luscombe R, Jeffries P, Ehrendorfer S, Heal C (2016). Volar locking distal radius plates show better short-term results than other treatment options: A prospective randomised controlled trial. World J Orthopedics.

[13] Boretto JG, Pacher N, Giunta D, Gallucci GL, Alfie V, De Carli P: Comparative clinical study of locking screws versus smooth locking pegs in volar plating of distal radius fractures. J Hand Surg EurVol 2014, 39(7):755-760. 10.1177/1753193413517806 24401740

[14] Weninger P, Dall'Ara E, Leixnering M, Pezzei C, Hertz H, Drobetz H, Redl H, Zysset P (2010). Volar fixed-angle plating of extra-articular distal radius fractures—a biomechanical analysis comparing threaded screws and smooth pegs. J Trauma Acute Care Surg.

[15] Willis AA, Kutsumi K, Zobitz ME, Cooney WP (2006). Internal fixation of dorsally displaced fractures of the distal part of the radius. A biomechanical analysis of volar plate fracture stability. J Bone Joint Surg Am.

[16] Soong M, Van Leerdam R, Guitton TG, Got C, Katarincic J, Ring D (2011). Fracture of the distal radius: risk factors for complications after locked volar plate fixation. J Hand Surg Am.

[17] Soong M, Earp BE, Bishop G, Leung A, Blazar P (2011). Volar locking plate implant prominence and flexor tendon rupture. J Bone Joint Surg Am.

[18] Toros T, Sugun TS, Ozaksar K (2013). Complications of distal radius locking plates. Injury.

[19] Plant CE, Parsons NR, Costa ML (2017). Do radiological and functional outcomes correlate for fractures of the distal radius?. Bone Joint J.

[20] Costa ML, Achten J, Parsons NR, Rangan A, Griffin D, Tubeuf S, Lamb SE (2014). Percutaneous fixation with Kirschner wires versus volar locking plate fixation in adults with dorsally displaced fracture of distal radius: randomised controlled trial. BMJ.

[21] Calfee RP, Adams AA (2012). Clinical research and patient-rated outcome measures in hand surgery. J Hand Surg Am.

[22] MacDermid JC, Richards RS, Donner A, Bellamy N, Roth JH (2000). Responsiveness of the short form-36, disability of the arm, shoulder, and hand questionnaire, patient-rated wrist evaluation, and physical impairment measurements in evaluating recovery after a distal radius fracture. J Hand Surg Am.

[23] Costa ML, Achten J, Plant C, Parsons NR, Rangan A, Tubeuf S, Yu G, Lamb SE (2015). UK DRAFFT: a randomised controlled trial of percutaneous fixation with Kirschner wires versus volar locking-plate fixation in the treatment of adult patients with a dorsally displaced fracture of the distal radius. Health Technol Assess.

[24] Mulders MA, Walenkamp MM, Goslings JC, Schep NW (2016). Internal plate fixation versus plaster in displaced complete articular distal radius fractures, a randomised controlled trial. BMC Musculoskelet Disord.

[25] Constand MK, MacDermid JC, Law M, Dal Bello-Haas V (2014). Patient-centered care and distal radius fracture outcomes: a prospective cohort study analysis. J Hand Ther.

[26] Bartl C, Stengel D, Bruckner T, Rossion I, Luntz S, Seiler C, Gebhard F (2011). Open reduction and internal fixation versus casting for highly comminuted and intra-articular fractures of the distal radius (ORCHID): protocol for a randomized clinical multi-center trial. Trials.

[27] Jupiter JB, Marent-Huber M (2009). Operative management of distal radial fractures with 2.4-millimeter locking plates. A multicenter prospective case series. J Bone Joint Surg Am.

[28] Walenkamp MM, de Muinck Keizer RJ, Goslings JC, Vos LM, Rosenwasser MP, Schep NW: The Minimum Clinically Important Difference of the Patient-rated Wrist Evaluation Score for Patients With Distal Radius Fractures. Clin Orthop Relat Res 2015, 473(10):3235-3241. 10.1007/s11999-015-4376-9 PMC4562929 26040969

[29] Goldhahn J, Beaton D, Ladd A, Macdermid J, Hoang-Kim A (2014). Recommendation for measuring clinical outcome in distal radius fractures: a core set of domains for standardized reporting in clinical practice and research. Arch Orthop Trauma Surg.

[30] Changulani M, Okonkwo U, Keswani T, Kalairajah Y (2008). Outcome evaluation measures for wrist and hand: which one to choose?. Int Orthop.

[31] MacDermid JC, Turgeon T, Richards RS, Beadle M, Roth JH (1998). Patient rating of wrist pain and disability: a reliable and valid measurement tool. J Orthop Trauma.

[32] Wilcke MK, Abbaszadegan H, Adolphson PY (2007). Patient-perceived outcome after displaced distal radius fractures: a comparison between radiological parameters, objective physical variables, and the DASH score. J Hand Ther.

[33] Obradovic M, Lal A, Liedgens H (2013). Validity and responsiveness of EuroQol-5 dimension (EQ-5D) versus Short Form-6 dimension (SF-6D) questionnaire in chronic pain. Health Qual Life Outcomes.

