# Supplementary material for: POWIFF- Prospective study of wrist internal fixation of fracture: A protocol for a single centre, superiority, randomised controlled trial to study the efficacy of the VRP (2.0) distal radius plate (Austofix) versus the VA-LCP (Depuy-Synthes) for distal radius fractures
Source: BMC Musculoskelet Disord. 2018 Apr 30;19:131. doi: 10.1186/s12891-018-2052-4 (PMC5928597; doi:10.1186/s12891-018-2052-4)
Supplement: Supplementary file 1 — Patient booklet and consent form with information sheet. (DOCX 49 kb) [file 12891_2018_2052_MOESM1_ESM.docx]

Participant Information Sheet & Consent form

POWIFF - Prospective Wrist Fracture Fixation Trial

Principal Investigator: Associate Professor Mark Rickman MD, FRACS (Tr & Orth)

Director of Orthopaedic Trauma, Royal Adelaide Hospital

Discipline of Orthopaedics and Trauma, University of Adelaide

Phone 82323065

Coinvestigator: Dr.V.D.Varghese, MBBS, MS (Orth)

Trauma Fellow, Royal Adelaide Hospital

Discipline of Orthopaedics and Trauma, University of Adelaide

Phone 0413400576

Coinvestigator: Mr. Peter Smitham, PhD, FRCS (Tr & Orth)

Trauma Fellow, Royal Adelaide Hospital

Discipline of Orthopaedics and Trauma, University of Adelaide

Phone 82323065

**Introduction**

We are inviting you to take part in a clinical research study which is a comparison of 2 different types of plates used to fix broken wrists.

Before you decide whether to participate, it is important for you to understand why this study is being undertaken and what it will involve.

Please take time to read the following information carefully and discuss it with your family or other advisors, if you wish.

Please ask if you would like additional information or there is anything that is not clear.

Take your time to consider whether or not you wish to take part.

What is this trial about?

Wrist fractures are common, and the majority of more severe ones are treated with surgery using plates and screws. We are carrying out a study looking at the effectiveness of a new plate used to fix wrist fractures. It is very similar to the plate that we usually use (including being made from the same metal), but with some important differences regarding how easy it is to put in, and how well it will fix the broken bones. Once the operation is over, as a patient you will not be able to tell which plate you have, and they will feel exactly the same. There is a possibility that the new plate will perform better, but we don’t know this and so we have to compare the two systems. There is no reason for us to think the new plate will be any worse than the present one.

Why have I been asked to participate?

You have broken your wrist, and your surgeon has already decided that you need an operation using this type of plate. We would therefore like to consider enrolling you into this trial.

What will happen if I agree to take part?

If you agree to take part, you will be randomly assigned to receive either the new plate or the standard one. Your operation will still take place in the normal way at the same time, by the same surgeon; the only difference will be which plate is used to fix the fracture.

After surgery we would normally review you in the clinic 3 times over the first 6 months, with clinical review and x-rays. For this study we would need to see you at 12 months after surgery for a review and an x-ray (which you will not receive any bill for). We will still see you at 2 weeks and 3 months, but the normal 6 weeks review will not happen in person unless there are problems – instead we will telephone you at this point. Your number of clinic visits and xrays will therefore be unchanged from our standard practise. In addition to clinical reviews and xrays, at 6 weeks, 3 months and 12 months from now we will fill out a scoring form with you, to assess the function of your wrist. This should take no more than 15 minutes each time, and the 6 weeks appointment can be done by telephone. The data will be stored on a password protected computer, and your personal data will not be part of this; your trial data (ie type of plate used, xrays, scores recorded etc) will be identified by a unique trial number given to all people who agree to take part.

You will not know which plate has been used, although we will tell you if you want to know, after the 12 month final assessment.

Are there any risks to me if I agree to take part?

There is no additional risk involved in the surgery or recovery process. You would need an extra x-ray at 12 months after surgery, which involves a small amount of radiation, but the risk associated with this is very small indeed. All operations carry some risk, and these will be discussed with you as part of your consent for the operation, but the risks associated with the new plate should be no different to the old one.

What will happen if I refuse to take part?

If you don’t want to take part in the trial then there is no problem with this. Your surgery will go ahead exactly as planned, and you will receive the standard plate that we have used before. There will be no change in your follow-up plans either.

What if I want to pull out of the study after surgery?

If you no longer wish to participate in the trial, then you are free to withdraw at any point. There will be no effect on your care as a result of this.

What are the potential benefits of this study?

You will not receive any direct benefits or payment for being in this study. We think that the new plate may offer benefits in terms of ease of use, as well as having better options for fixing the fracture. This could lead to shorter operation times, and better long term outcomes from the injury.

Who is organising and funding the research?

This study is being organised and run by Associate Professor Mark Rickman, as part of the department of Orthopaedics & Trauma at the Royal Adelaide Hospital. There is no specific funding involved, although a small amount of funding is required for some aspects (e.g. data analysis) which will be funded from the orthopaedic trauma research fund.

All research in medicine is looked at by independent group of people, called a Research Ethics Committee, to protect your interests. This study has been reviewed and given favourable opinion by the ethics committee. Your medical records for information related to your operation and any post-operative events will be required by the research team.

All the information collected during the study will be kept confidential. Data will be held on a secure database in the Royal Adelaide Hospital, protected from unauthorised access.

**Confidentiality and Data Security**

On the database, you will be identified by a unique study number, date of birth, date of operation and surgeon who performed your operation. Only the surgeon, members of the research team and auditors will be able to identify participant’s names. All parties are bound by strict confidentiality guidelines under the Australian Data Protection Laws.

If you agree to participate you will be asked to grant consent for our research team to access your medical notes for data entry and to auditors for the purpose of verifying accuracy of data entered. Data collected will include standard medical data about you (such as age, sex, weight, and medical history), the details of your operation, and the results of the forms that you fill in over the years of this study. Information from your X-rays will also be collected. The data will be kept in a secure electronic form on a password protected hospital computer, and will be destroyed 15 years after the completion of the study.

In addition to the processes described above, data may otherwise be discoverable through processes of law or for assessing compliance with research procedures.

You have a right to access the information collected and stored by researchers about you. You also have a right to request that any information with which you disagree be corrected.

You have a right to ask that any stored specimens be destroyed but should be aware that data which has already been derived from those specimens may not be able to be destroyed.

What will happen to the results?

Whatever the trial shows, we plan to publish the data as a paper in the medical literature, as well as present it at local and national meetings to disseminate the findings. No patients would be identified in any of these however, and only total numbers of patients and outcomes will be shown.

Who do I ask if I have more questions?

If you have urgent questions, you can ask either the person who gave you this form, or your treating doctors. In addition, for less urgent questions you can ask the study co-ordinator A/Professor Mark Rickman, who can be contacted via his secretary on 08 8222 5535

Prospective Wrist Fracture Fixation Trial **
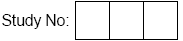
**

**PATIENT CONSENT FORM**

1. I confirm that I agree to take part in this study as described to me and that I was given the opportunity to ask all of the questions I had concerning my treatment and participation and that they were all answered to my satisfaction.
2. I also confirm that I have read and understood the patient information sheet and I have had the opportunity to discuss the patient information provided for me with members of my family and/or friends.
3. I understand that my participating is voluntary. I also understand that I am able to withdraw from this study at any time without giving any reason and without it compromising my future treatment or legal rights.
4. I agree to complete the relevant questionnaires and return for follow-up visits when necessary.
5. I give my consent for appropriately qualified persons to have access to my medical records relevant to the data concerning my operation and participation in the study. I understand that this will only be necessary for assistance with the monitoring of the quality of data being collected during the study and to help with any analysis of the data. I understand that these people are bound by strict guidelines of professional conduct to maintain the confidentiality of any information they review.
6. I understand that data collected about me during this follow-up study will be kept in paper documents and on a computer database, and that all data management conforms to the Data Protection Act 1998. I also understand that this data will be used in the preparation of research publications for this study only, and that at no time will I be identifiable personally within any of these publications without my prior written consent.
7. I understand that if I withdraw or become unable to complete the study on medical grounds that data gathered prior to that time point may still be used for this study

Patient Name ……………………………………………

Signature ………………………………………………… Date ………………

Consent Taken By ……………………………………… Role………………

Signature ………………………………………………… Date ………………

Surgeon Name……………………………………………

Surgeon Signature……………………………………….. Date………………
